# Supplementary material for: Peronosporales Species Associated with Strawberry Crown Rot in the Czech Republic
Source: J Fungi (Basel). 2022 Mar 26;8(4):346. doi: 10.3390/jof8040346 (PMC9024537; doi:10.3390/jof8040346)
Supplement: Supplementary file 1 [file jof-08-00346-s001.zip › jof-1627628-supplementary.pdf]

Table S1. The list of all isolates of Peronosporales found out in strawberry fields in the Czech Republic. The names of sampled localities and NCBI accession numbers of ITS sequences of all isolates are given.

Plantation ID	Location of strawberry plantation	Species ID	Isolate ID	NCBI GenBank accession number
1	Blansko	*Pythium dissotocum* complex	(19)18_01_6b	OK257542
2	Praha	*Pythium dissotocum* complex	18_02_1a	OK257549
2	Praha	*Phytophthora cactorum*	18_02_1b	MW193099
2	Praha	*Phytophthora cactorum*	18_02_3	OK257672
3	Praha	*Phytophthora cactorum*	17_03_10	MW193100
3	Praha	*Phytophthora cactorum*	17_03_11	OK257589
3	Praha	*Phytophthora cactorum*	17_03_12	MW646877
3	Praha	*Phytophthora cactorum*	17_03_13	OK257590
3	Praha	*Phytophthora cactorum*	17_03_23	OK257591
3	Praha	*Phytophthora cactorum*	17_03_24	MW646878
3	Praha	*Phytophthora cactorum*	17_03_5	OK257587
4	Praha	*Phytophthora cactorum*	17_04_10	MW646879
4	Praha	*Phytophthora cactorum*	17_04_12	MW193106
4	Praha	*Phytophthora cactorum*	17_04_14	OK257599
4	Praha	*Phytophthora cactorum*	17_04_1a	OK257592
4	Praha	*Phytophthora cactorum*	17_04_2	OK257593
4	Praha	*Phytophthora cactorum*	17_04_3	OK257594
4	Praha	*Phytophthora cactorum*	17_04_5	OK257595
4	Praha	*Phytophthora cactorum*	17_04_7a	OK257596
4	Praha	*Phytophthora cactorum*	17_04_8	OK257597
4	Praha	*Phytophthora cactorum*	17_04_9	OK257598
5	Havlíčkův Brod	–	No isolate	-
7	Předměřice nad Jizerou	*Globisporangium irregulare*	(19)18_07_1	OK257580
7	Předměřice nad Jizerou	*Phytopythium vexans*	17_07_1	MW193096
7	Předměřice nad Jizerou	*Pythium perplexum*	17_07_10	OK257585
7	Předměřice nad Jizerou	*Phytophthora cactorum*	17_07_12a	OK257601
7	Předměřice nad Jizerou	*Pythium dissotocum* complex	17_07_17	OK257543
7	Předměřice nad Jizerou	*Phytopythium mercuriale*	17_07_22	MW646900
7	Předměřice nad Jizerou	*Phytophthora cactorum*	17_07_23	OK257600
7	Předměřice nad Jizerou	*Phytophthora cactorum*	17_07_25	MW646883
7	Předměřice nad Jizerou	*Phytophthora cactorum*	17_07_27a	MW646884
7	Předměřice nad Jizerou	*Phytophthora citrophthora*	17_07_9	MW193107
7	Předměřice nad Jizerou	*Pythium intermedium*	18_07_11	OK257571
7	Předměřice nad Jizerou	*Phytophthora cactorum*	18_07_12a	MW646885
7	Předměřice nad Jizerou	*Pythium intermedium*	18_07_12c	OK257572
7	Předměřice nad Jizerou	*Pythium dissotocum* complex	18_07_13	OK257553
7	Předměřice nad Jizerou	*Phytophthora cactorum*	18_07_14	OK257669
7	Předměřice nad Jizerou	*Phytopythium citrinum*	18_07_15	OK257573
7	Předměřice nad Jizerou	*Phytophthora cactorum*	18_07_2_S1	MW646889
7	Předměřice nad Jizerou	*Pythium dissotocum* complex	18_07_2_S10	OK257550
7	Předměřice nad Jizerou	*Pythium dissotocum* complex	18_07_3a	OK257551
7	Předměřice nad Jizerou	*Phytophthora cactorum*	18_07_6	OK257668
7	Předměřice nad Jizerou	*Phytopythium vexans*	18_07_9	OK257569
8	Sedlčánky	*Phytophthora cactorum*	17_08_10	OK257603
8	Sedlčánky	*Phytophthora cactorum*	17_08_17b	OK257604
8	Sedlčánky	*Pythium dissotocum* complex	17_08_3	OK257544
8	Sedlčánky	*Phytophthora cactorum*	17_08_6	OK257602
9	Oskořínek	*Phytophthora cactorum*	17_09_12	OK257605

Plantation ID	Location of strawberry plantation	Species ID	Isolate ID	NCBI GenBank accession number
9	Oskořínek	*Phytophthora cactorum*	17_09_14a	OK257606
10	Břežany II	*Pythium nodosum*	18_10_10	OK257564
10	Břežany II	*Phytophthora cactorum*	18_10_11	MW646888
10	Břežany II	*Phytophthora cactorum*	18_10_12	MW646895
10	Břežany II	*Pythium dissotocum* complex	18_10_12a	OK257555
10	Břežany II	*Pythium dissotocum* complex	18_10_13	OK257556
10	Břežany II	*Phytophthora cactorum*	18_10_14a	MW193116
10	Břežany II	*Pythium nodosum*	18_10_14b	OK257565
10	Břežany II	*Phytophthora cactorum*	18_10_16	OK257673
10	Břežany II	*Phytophthora cactorum*	18_10_17a	MW646886
10	Břežany II	*Pythium nodosum*	18_10_17b	OK257566
10	Břežany II	*Phytophthora cactorum*	18_10_18c	MW646887
10	Břežany II	*Pythium dissotocum* complex	18_10_5a	OK257554
10	Břežany II	*Pythium dissotocum* complex	18_10_7a	OK257552
11	Domašín	*Phytophthora cactorum*	17_11_16	OK257608
11	Domašín	*Phytophthora cactorum*	17_11_17	OK257609
11	Domašín	*Phytophthora cactorum*	17_11_19	MW646891
11	Domašín	*Phytophthora cactorum*	17_11_3	OK257607
12	Plzeň	*Phytophthora cactorum*	17_12_10	OK257616
12	Plzeň	*Phytophthora cactorum*	17_12_12	OK257617
12	Plzeň	*Phytopythium vexans*	17_12_13	OK257568
12	Plzeň	*Phytophthora cactorum*	17_12_16	OK257618
12	Plzeň	*Phytophthora cactorum*	17_12_17a	OK257619
12	Plzeň	*Phytophthora cactorum*	17_12_18a	OK257620
12	Plzeň	*Phytophthora cactorum*	17_12_1b	MW646880
12	Plzeň	*Phytophthora cactorum*	17_12_20	MW193108
12	Plzeň	*Phytophthora cactorum*	17_12_23	OK257621
12	Plzeň	*Phytophthora cactorum*	17_12_24	OK257622
12	Plzeň	*Phytophthora cactorum*	17_12_25	OK257623
12	Plzeň	*Phytophthora cactorum*	17_12_26	OK257624
12	Plzeň	*Phytophthora cactorum*	17_12_27	OK257625
12	Plzeň	*Phytophthora cactorum*	17_12_28	OK257626
12	Plzeň	*Pythium dissotocum* complex	17_12_29	OK257545
12	Plzeň	*Phytophthora cactorum*	17_12_3	OK257610
12	Plzeň	*Phytophthora cactorum*	17_12_30	OK257627
12	Plzeň	*Phytophthora cactorum*	17_12_31	OK257628
12	Plzeň	*Phytophthora cactorum*	17_12_4	OK257611
12	Plzeň	*Phytophthora cactorum*	17_12_5a	OK257612
12	Plzeň	*Phytophthora cactorum*	17_12_6a	MW646882
12	Plzeň	*Phytophthora cactorum*	17_12_7	OK257613
12	Plzeň	*Phytophthora cactorum*	17_12_8	OK257614
12	Plzeň	*Phytophthora cactorum*	17_12_9	OK257615
15	Holešov	*Phytophthora cactorum*	17_15_1	OK257629
15	Holešov	*Phytophthora cactorum*	17_15_10	MW193114
15	Holešov	*Phytophthora cactorum*	17_15_4a	OK257630
15	Holešov	*Phytophthora cactorum*	17_15_8	OK257631
18	Brno	*Phytophthora cactorum*	17_18_26	OK257632
23	Novákovice	*Phytophthora cactorum*	17_23_16	OK257639
23	Novákovice	*Phytophthora cactorum*	17_23_19	MW193104
23	Novákovice	*Phytophthora cactorum*	17_23_1a	OK257633
23	Novákovice	*Phytophthora cactorum*	17_23_3a	OK257634

Plantation ID	Location of strawberry plantation	Species ID	Isolate ID	NCBI GenBank accession number
23	Novákovice	*Phytophthora cactorum*	17_23_4a	OK257635
23	Novákovice	*Phytophthora cactorum*	17_23_7	OK257636
23	Novákovice	*Phytophthora cactorum*	17_23_8	OK257637
23	Novákovice	*Phytophthora cactorum*	17_23_9	OK257638
24	Lotouš	*Phytophthora cactorum*	17_24_12	OK257644
24	Lotouš	*Phytophthora cactorum*	17_24_19	OK257645
24	Lotouš	*Phytophthora cactorum*	17_24_20	OK257646
24	Lotouš	*Phytophthora cactorum*	17_24_26	OK257647
24	Lotouš	*Phytophthora cactorum*	17_24_3	OK257640
24	Lotouš	*Phytophthora cactorum*	17_24_4	OK257641
24	Lotouš	*Phytopythium vexans*	17_24_59	MW193115
24	Lotouš	*Pythium conidiophorum*	17_24_5b	OK257586
24	Lotouš	*Phytophthora cactorum*	17_24_5c	OK257656
24	Lotouš	*Pythium dissotocum* complex	17_24_6	OK257546
24	Lotouš	*Phytophthora cactorum*	17_24_7	OK257642
24	Lotouš	*Phytophthora cactorum*	17_24_8a	OK257643
26	Sedlčánky	*Phytophthora cactorum*	17_26_12	MW193094
26	Sedlčánky	*Phytophthora cactorum*	17_26_14	OK257649
26	Sedlčánky	*Phytophthora cactorum*	17_26_3	OK257648
26	Sedlčánky	*Pythium dissotocum* complex	17_26_6	OK257547
26	Sedlčánky	*Phytopythium litorale*	17_26_8	MW193098
26	Sedlčánky	*Pythium dissotocum* complex	17_26_9	OK257548
27	Turnov	*Pythium dissotocum* complex	17_27_2	MW193105
28	Přelovice	*Phytophthora cactorum*	19_28_10	OK257674
28	Přelovice	*Pythium mamillatum*	19_28_12b	OK257583
28	Přelovice	*Phytophthora bilorbang*	19_28_2	OK257676
28	Přelovice	*Globisporangium ultimum*	19_28_3a	MW193117
28	Přelovice	*Globisporangium irregulare*	19_28_3c	OK257579
28	Přelovice	*Globisporangium irregulare*	19_28_4	OK257581
28	Přelovice	*Pythium dissotocum* complex	19_28_5	OK257557
28	Přelovice	*Pythium mamillatum*	19_28_6b	OK257582
28	Přelovice	*Phytophthora lacustris*	19_28_7	MW646898
28	Přelovice	*Pythium dissotocum* complex	19_28_8a	OK257558
28	Přelovice	*Pythium dissotocum* complex	19_28_9b	OK257559
30	Svádov	*Phytophthora cactorum*	17_30_12a	OK257653
30	Svádov	*Phytophthora cactorum*	17_30_13	OK257654
30	Svádov	*Phytophthora cactorum*	17_30_18	OK257655
30	Svádov	*Phytophthora cactorum*	17_30_3	OK257650
30	Svádov	*Phytophthora cactorum*	17_30_6	MW646894
30	Svádov	*Phytophthora cactorum*	17_30_8	OK257651
30	Svádov	*Phytophthora cactorum*	17_30_9	OK257652
31	Němčičky	*Pythium nodosum*	17_31_6	OK257563
33	Šakvice	*Globisporangium ultimum*	18_33_12	OK257577
33	Šakvice	*Phytophthora cactorum*	18_33_3	MW646893
33	Šakvice	*Pythium heterothallicum*	18_33_3b	OK257575
34	Poděbrady	*Pythium torulosum*	17_34_4	OK257561
34	Poděbrady	*Phytophthora cactorum*	17_34_7	OK257657
37	Brožany	*Phytophthora cactorum*	17_37_10	OK257659
37	Brožany	*Phytophthora cactorum*	17_37_11	OK257660
37	Brožany	*Phytophthora cactorum*	17_37_15	OK257661
37	Brožany	*Phytophthora cactorum*	17_37_7a	OK257658

Plantation ID	Location of strawberry plantation	Species ID	Isolate ID	NCBI GenBank accession number
38	Chlumec	–	No isolate	-
39	Vrkoč	*Globisporangium ultimum*	(19)18_39_6a	MW193111
39	Vrkoč	*Pythium aphanidermatum*	(19)18_39_6b	MW193112
41	Louny	-	No isolate	-
42	Starý Kolín	*Pythium torulosum*	18_42_16	OK257562
42	Starý Kolín	*Phytophthora cactorum*	19_42_10a	MW193113
42	Starý Kolín	*Pythium dissotocum* complex	19_42_15	OK257560
42	Starý Kolín	*Phytophthora cactorum*	19_42_17a	OK257671
42	Starý Kolín	*Pythium rostratifingens*	19_42_7b	OK257584
43	Kouřim	*Pythium heterothallicum*	(19)18_43_1	MW646902
43	Kouřim	*Pythium salpingophorum*	(19)18_43_9	OK257588
44	Stebno	*Phytophthora cactorum*	17_44_12	OK257662
45	Veselá	*Phytophthora cactorum*	17_45_1a	OK257663
45	Veselá	*Phytophthora cactorum*	17_45_1b	OK257664
48	Lovčičky	*Phytopythium montanum*	18_48_1	OK257578
50	Pojedy	*Pythium nodosum*	17_50_1	OK257567
51	Smrkov	–	No isolate	-
53	Mokrá	*Phytophthora cactorum*	17_53_3	OK257665
55	Malochyně	–	No isolate	-
56	Svijanský Újezd	–	No isolate	-
57	Vraňany nad Vltavou	*Phytophthora citrophthora*	17_57_1P	MW646897
57	Vraňany nad Vltavou	*Phytophthora cactorum*	17_57_F1	OK257666
57	Vraňany nad Vltavou	*Phytopythium vexans*	18_57_64	OK257570
58	Sobíňov	–	No isolate	-
60	Lhota pod Libčany	*Phytophthora cactorum*	17_60_25	OK257667
60	Lhota pod Libčany	*Phytophthora cactorum*	17_60_26	MW193095
62	Hradec Králové	*Pythium heterothallicum*	(19)18_62_5b	OK257576
62	Hradec Králové	*Pythium intermedium*	(19)18_62_9a	OK257574
62	Hradec Králové	*Phytophthora cactorum*	18_62_9	OK257670
99	Josefínská Huť	*Phytophthora pluvivora*	17_99_1	MW646899
99	Josefínská Huť	*Phytophthora cryptogea*	17_99_2	OK257675
